# Une tumeur du vagin à ne pas méconnaitre, l'adénocarcinome mésonephrique: à propos d'un cas et revue de la literature

**DOI:** 10.11604/pamj.2015.21.126.5754

**Published:** 2015-06-15

**Authors:** Bennani Amal, El Fatemi Hind, Saadi Hanane, Rabhi Hayat, Moumna Kaoutar, Banani Abdelaziz, Harmouch Taoufik, Amarti Affaf

**Affiliations:** 1 Service d'Anatomie Pathologique, CHU Hassan II, Fès, Maroc; 2 Service de Gynécologie Obstétrique, CHU Hassan II, Fès, Maroc

**Keywords:** Mésonéphrique, adénocarcinome, vagin, pancytokératine, CD10, Mesonephric, adenocarcinoma, vagina, pancytokeratins, CD10

## Abstract

L'adénocarcinome mésonéphrique du vagin est une tumeur maligne extrêmement rare avec uniquement trois cas publiés dans la littérature jusqu’à maintenant. Il dérive des reliquats embryonnaires des canaux mésonéphriques au niveau du vagin. Nous rapportons un cas d'adénocarcinome mésonéphrique du vagin survenant chez une femme de 50 ans, et révélé par une masse polyploïde du vagin. L'IRM a montré un envahissement du périnée et de la branche inférieure du pubis. L’étude anatomo-pathologique était en faveur d'un adénocarcinome mésonéphrique dont les cellules tumorales expriment la pancytokératine et le CD10. Elles ne sont pas marquées par les anticorps anti récepteurs ostrogéniques et progestatifs. La patiente a été adressée pour radiothérapie avant la prise en charge chirurgicale. Les auteurs soulignent à travers cette observation les aspects étiopathogéniques, histologiques et thérapeutiques de cette tumeur rare.

## Introduction

L'adénocarcinome mésonéphrique du vagin est une tumeur extrêmement rare avec quelques cas publiés dans la littérature. Le diagnostic différentiel se pose avec les autres tumeurs agressives du vagin. Plusieurs éléments restent inconnus concernant l’évolution, le pronostic et les stratégies thérapeutiques proposées pour cette tumeur.

## Patient et observation

**Figure 1:**
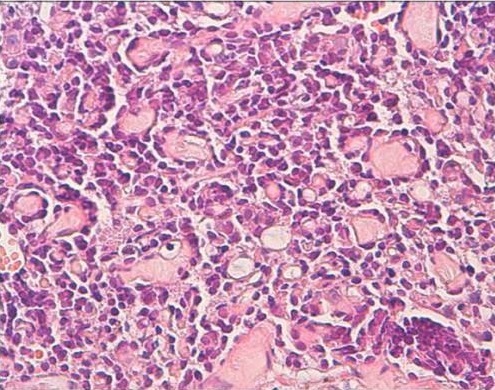
Adénocarcinome mésonephrique bien différencié fait de structures glandulaires comportant dans leur lumière une substance éosinophile avec des atypies cytonucléaires

**Figure 2:**
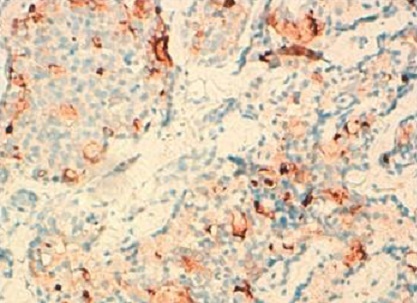
Positivité focale des cellules tumorales pour le CD10

**Figure 3:**
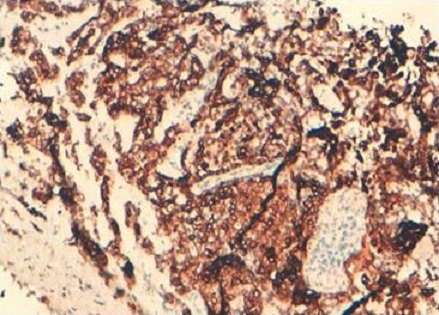
Positivité des cellules tumorales pour le CK7

Une femme de 50 ans, consulte pour des métrorragies depuis 2 ans. L´examen clinique a montré un col aspiré avec disparition des culs de sac vaginaux avec présence d'une lésion de la paroi latérale du vagin saignant au contact de 4 cm. L'IRM a objectivé un processus malin du vagin envahissant le périnée et la branche inférieure du pubis ce qui permet de la classer au stade III. Une biopsie a été réalisée. L’étude histologique de la biopsie de cette masse a montré que le chorion vaginal était le siège d'une prolifération tumorale d'architecture tubulaire (Figure 1). Les lumières étaient comblées par un matériel éosinophile. Les cellules présentaient des atypies modérées et quelques mitoses. Une image d'engainement périnerveux était observée. Devant cet aspect le diagnostic d'adénocarcinome mésonéphrique infiltrant du vagin a été porté. Les cellules tumorales étaient positives pour les anticorps anti-pancytokératine, anti-CD10 (Figure 2), anti-CK7 (Figure 3), et négatif pour les récepteurs oestrogéniques et progestatifs. La décision thérapeutique a été de réaliser une curiethérapie avant la chirurgie.

## Discussion

L'adénocarcinome mésonéphrique du vagin est extrêmement rare. Il se développe à partir des restes des conduits mésonéphriques (canal de wolff). Ces conduits cheminent le long du ligament large, des parois latérales de l'utérus et du col utérin et arrivent au niveau du vagin où ils sont appelés conduit de Gartner. Ils sont retrouvés sur 20% des cols enlevés dans le carde d'hystérectomies systématiques. Des cas de tumeurs mésonephriques ont été rapportés dans le corps utérin [1], le col utérin [2, 3], le ligament large [4], la vessie [5], l´urètre [6], et le diverticule urétral [7]. Cependant, une bonne partie de ces tumeurs, qui étaient auparavant classées comme des adénocarcinomes mésonephriques sont actuellement classés comme des carcinomes à cellules claires de type Müller. Ainsi, l´incidence réelle des adénocarcinomes mésonéphriques est difficile à déterminer en partie à cause de cette confusion histologique dans la littérature. Actuellement, trois cas bien documentés de l´adénocarcinome découlant de restes de conduits mésonéphrotiques au niveau du vagin ont été rapportés [8–10]. Par Ailleurs, il existe peu de données publiées sur la présentation clinique et l’évolution. L’âge moyen des patientes lors du diagnostic est de 50 ans. Elles se présentent souvent avec des saignements vaginaux anormaux. Dans la plupart des cas, l´examen gynécologique révèle une masse qui peut être exophytique ou endophytique. Dans notre cas, la tumeur était située au niveau de la paroi vaginale droite. Histologiquement, les adénocarcinomes mésonephriques peuvent être purs ou associés à une composante à cellules fusiforme. L´aspect le plus fréquent se présente sous forme de structures tubulaires de taille variable bordées par une ou plusieurs couches de cellules cylindriques. La lumière de ces structures comporte une substance PAS positive. Des études immunohistochimiques récentes réalisées dans l´adénocarcinome mésonephrique du col utérin [1, 2] ont rapporté que les marqueurs épithéliaux y compris pancytokeratin, CK7, CAM 5.2, et l´EMA sont constamment exprimés par les cellules tumorales. La vimentine est positive dans 70% des 11 cas, la calrétinine dans 88%, et les récepteurs androgéniques dans 33% des cas, l'expression du CD10 dans un adénocarcinome mésonéphrique du corps utérin a été également rapportée [1]. L'anticorps Monoclonal CK20, ACE, les récepteurs oestrogéniques et progestatifs ont été négatifs. Notre tumeur a montré une positivité pour la pancytokeratin, CK7, EMA, CD10, et une négativité pour CK20, ER et PR.

Le diagnostic différentiel de l´adénocarcinome mésonéphrique inclut le carcinome à cellules claires, le carcinome séreux, les tumeurs mixtes malignes de Müller (MMMT), l´hyperplasie de Wolff, et l´adénocarcinome endométrioïde. Le problème de diagnostic différentiel se pose essentiellement dans la littérature entre l´adénocarcinome et le carcinome mésonéphrique à cellules claires. Toutefois, le carcinome à cellules claires montre généralement des degrés variables de structures kystique, papillaire, ou solide, avec deux types de cellules peuvent retrouvées: les cellules claires à cytoplasme abondant autour d´un noyau central et les cellules cloutés avec une moindre quantité de cytoplasme granuleux opaque et un noyau excentré. Le PAS est positive à la fois dans les sécrétions intraluminales et les globules intra cytoplasmiques. Par ailleurs, la distinction entre un carcinome mésonéphrique et simple hyperplasie peut être difficile parce que la majorité des carcinomes se développent sur une hyperplasie des conduits mésonephriques qui est diffuse. Néanmoins, il faut noter que, contrairement à l'hyperplasie, le carcinome n´a pas une architecture lobulaire et les noyaux apparaissent cytologiquement malins. Il est possible également que l´adénocarcinome mésonephrique peut-être sous-diagnostiquée en raison de sa ressemblance avec l'adénocarcinome endométrioïde d'où l'intérêt des études immunohistochimiques dans les cas difficiles (récepteurs hormonaux). Les adénocarcinomes mésonéphriques biphasiques avec une composante sarcomatoïde ont été rapportés, ce qui peut être confondue avec les MMMT [11, 12]. Cependant, dans notre cas, le stroma était bénin, écartant ainsi cette possibilité. La rareté globale de l´adénocarcinome mésonéphrique du vagin rend difficile l’étude de son évolution et son pronostic. Cependant, cette tumeur semble avoir un comportement plus indolent que les tumeurs malignes d'origine mullerienne [11]. Peu de données sont publiées dans la littérature concernant la prise en charge optimale de ce type de tumeur [10].

## Conclusion

L'adénocarcinome mésonephrique du vagin est une entité rare. Le diagnostic est souvent aisé en cas d'hyperplasie des restes mésonephriques associée. Sa prise en charge rejoint celles des autres tumeurs agressives du vagin.
